# Comparative diagnostic performance of plasma microbial cell-free DNA sequencing versus amplicon-based blood next-generation sequencing in suspected bloodstream or endovascular infection: a single-center study

**DOI:** 10.1017/ash.2026.10337

**Published:** 2026-03-27

**Authors:** Mayar Al Mohajer, Todd Lasco

**Affiliations:** 1 Nuffield Department of Primary Health Care and Science, University of Oxford, Oxford, UK; 2 Department of Medicine, Baylor College of Medicine, Houston, TX, USA; 3 Department of Pathology, Baylor College of Medicine, Houston, TX, USA

## Abstract

We compared plasma microbial cell-free DNA sequencing (mcfDNAseq) with an amplicon-based blood next-generation sequencing (NGS) assay for 10 patients with suspected bloodstream or endovascular infection. Plasma testing detected pathogens in eight (seven clinically meaningful), whereas amplicon testing was negative in all. Plasma mcfDNAseq provided higher test yield and overall sensitivity.

## Introduction

Bloodstream and endovascular infections require early identification of the causative organism to guide targeted antimicrobial therapy and source control.^1^ Standard evaluation includes blood cultures and syndrome-directed imaging (eg, echocardiography) and, when cultures are negative, targeted serologic or molecular testing for fastidious pathogens.^2,3^ Culture-negative presentations, including blood culture-negative endocarditis, are common after antibiotic exposure and are associated with worse outcomes.^2–4^

Plasma microbial cell-free DNA sequencing (mcfDNAseq) detects microbial DNA fragments in plasma and may identify pathogens when cultures are unrevealing.^5,6^ Amplicon-based blood next-generation sequencing (NGS) assays amplify conserved loci across selected taxa, but performance in low-biomass or intermittently bacteremic syndromes is uncertain.^3^ We compared these diagnostic approaches in hospitalized patients who underwent paired testing for suspected bloodstream or endovascular infection.

## Methods

### Design and setting

We conducted a retrospective, single-center review of hospitalized patients evaluated for suspected bloodstream and/or endovascular infection.

### Patients and inclusion criteria

We included consecutive inpatients with paired plasma (mcfDNAseq; Karius Test®) and an amplicon-based blood NGS assay (MicroGenDX) ordered within 24 h during August-October 2025, when paired ordering began at our institution. Each patient contributed one paired-testing episode.

### Index tests and data collection

For each episode, we abstracted molecular test results from the electronic health record and classified each assay as positive (at least 1 organism reported) or negative (no organism detected). Organisms reported by plasma mcfDNAseq were recorded as provided by the performing laboratory. Viral co-detections were captured descriptively, given differences in assay scope between platforms.

### Reference standard (clinical adjudication)

Because culture is an imperfect gold standard in suspected endovascular infection, infectious diseases clinician adjudication served as the reference standard. Adjudicators reviewed the complete clinical record (syndrome, imaging, blood cultures/other microbiology, and ancillary serologic or molecular diagnostics) to determine whether infection was present and whether organisms reported were clinically meaningful versus likely contaminant/incidental.’

### Outcomes

The primary outcome was assay concordance, defined as both assays positive or both assays negative for a given episode. Secondary outcomes included diagnostic classification (true/false positive or negative) versus clinical adjudication and descriptive organism patterns.

### Statistical analysis

We summarized the results descriptively. Using clinical adjudication as the reference, we calculated sensitivity and specificity for each assay with exact (Clopper–Pearson) 95% CIs.

### Ethics

This work was approved by the institutional review board at Baylor College of Medicine.

## Results

Ten patients contributed 10 paired-testing episodes. Overall concordance between assays was 2/10 (20%), and all concordant pairs were dual negative; there were no dual-positive pairs. Patient-level paired results are summarized in Table 1.

**Table 1.** Per-patient paired results for mcfDNAseq versus amplicon-based blood NGS (*n* = 10)

Patient	Plasma mcfDNAseq organism(s)	Amplicon blood NGS	Adjudication (mcfDNAseq)	Adjudication (amplicon)
P01	*Coxiella burnetii*	Negative	True positive	False negative
P02	*Pseudomonas aeruginosa*; cytomegalovirus	Negative	True positive	False negative
P03	*Legionella gormanii*; herpes simplex virus type 1	Negative	True positive	False negative
P04	*Enterococcus faecium*	Negative	True positive	False negative
P05	*Candida albicans*	Negative	True positive	False negative
P06	*Enterobacter cloacae*; *Escherichia coli*; *Klebsiella pneumoniae*	Negative	True positive	False negative
P07	*Candida parapsilosis*	Negative	True positive	False negative
P08	*Staphylococcus epidermidis*	Negative	False positive	True negative
P09	No organism detected	Negative	True negative	True negative
P10	No organism detected	Negative	True negative	True negative

mcfDNAseq, microbial cell-free DNA sequencing; NGS, next-generation sequencing.

Plasma mcfDNAseq was positive in 8/10 (80%) and negative in 2/10 (20%). Of the 8 positive mcfDNAseq results, 7/8 were adjudicated clinically meaningful (true positives), and 1/8 was adjudicated a contaminant (*Staphylococcus epidermidis*, false positive).

Amplicon-based blood NGS was negative in 10/10 (100%), including in all adjudicated infections, yielding 0% positivity in this cohort.

Clinically meaningful detections by plasma mcfDNAseq included *Coxiella burnetii*; *Pseudomonas aeruginosa* (with cytomegalovirus co-detection); *Legionella gormanii* (with herpes simplex virus type 1 co-detection); and polymicrobial detections involving *Candida* spp., *Enterococcus faecium*, *Enterobacter cloacae*, *Escherichia coli*, and *Klebsiella pneumoniae*. Viral co-detections reflected the broader scope of plasma mcfDNA testing relative to the bacterial/fungal amplicon assay.

Using clinical adjudication as the reference standard (infection present *n* = 7; infection absent *n* = 3): mcfDNAseq sensitivity was 100% (7/7; 95% CI 59–100) and specificity 67% (2/3; 95% CI 9–99). Amplicon-based NGS sensitivity was 0% (0/7; 95% CI 0–41) and specificity 100% (3/3; 95% CI 29–100).

## Discussion

In this single-center inpatient series of paired molecular testing, plasma mcfDNAseq produced substantially higher diagnostic yield than an amplicon-based blood NGS assay: 8/10 plasma tests were positive (7 clinically meaningful) whereas all amplicon tests were negative. Rapid organism identification is central to management of endovascular infection, and culture-negative presentations remain common, often after antibiotic exposure, or with fastidious organisms.^1–3^

Detected pathogens were clinically plausible for culture-negative or diagnostically complex endovascular syndromes. Notably, *C. burnetii*—a classic cause of culture-negative endocarditis typically diagnosed by serology—is emphasized in diagnostic frameworks.^2,3^ Culture-negative endocarditis has been associated with worse outcomes than culture-positive disease, reinforcing the importance of establishing an etiology when possible.^4^

These findings are consistent with prior analytic validation and clinical experience showing that plasma mcfDNA sequencing can detect a broad range of pathogens, including organisms that may be missed by conventional culture.^5^ Multicenter real-world studies suggest that plasma mcfDNA testing can be clinically impactful when used in appropriately selected patients.^6^

The lack of yield from amplicon-based blood NGS in our cohort may reflect differences in analyte (cell-free vs cell-associated nucleic acids), assay target scope, and low microbial burden in intermittently bacteremic syndromes. Although confirmatory studies are needed, these results suggest that concurrent ordering of amplicon-based blood NGS alongside plasma mcfDNAseq may add limited value in similar inpatient evaluations.

From an antimicrobial stewardship perspective, plasma mcfDNAseq may support earlier pathogen-directed therapy in culture-negative or complex endovascular infection, but results require clinical correlation. The single adjudicated falsepositive (*S. epidermidis*) highlights the need to interpret potential contaminants and incidental detections cautiously.^5,6^ Viral co-detections should similarly be interpreted in the context of syndrome and host factors, as detection does not necessarily indicate causation or need for therapy.^6^

This study is limited by a small sample size and a short accrual window, a retrospective single-center design, and selection bias because inclusion required paired testing. Clinical adjudication is an imperfect reference standard, and we did not assess turnaround time, treatment changes, costs, or outcomes.

In conclusion, plasma mcfDNAseq provided greater clinically actionable yield than an amplicon-based blood NGS assay and appeared highly sensitive for adjudicated infections; when a broad blood-based molecular test is indicated, plasma mcfDNAseq may be preferred, with stewardship-guided interpretation to mitigate falsepositive and incidental results.

## Data Availability

Deidentified data are available from the corresponding author upon reasonable request, subject to institutional policies.
